# Dynamic Interplay of Epithelial–Mesenchymal and Mesenchymal–Epithelial Transitions in Cochlear Development and Disease: Molecular Mechanisms and Therapeutic Implications

**DOI:** 10.3390/biomedicines13123115

**Published:** 2025-12-18

**Authors:** Jingyi Zhu, Sihan Huang, Jifang Zhang, Tianyu Gong, Zhongyuan Fei, Penghui Chen, Shule Hou, Jun Yang

**Affiliations:** 1 Department of Otorhinolaryngology-Head and Neck Surgery, Xinhua Hospital, Shanghai Jiao Tong University School of Medicine, Shanghai 200082, Chinacph_shsmu@163.com (P.C.); 2 Ear Institute, Shanghai Jiaotong University School of Medicine, Shanghai 200082, China; 3 Shanghai Key Laboratory of Translational Medicine on Ear and Nose Diseases, Shanghai 200082, China

**Keywords:** cochlea, epithelial–mesenchymal transition, mesenchymal–epithelial transition, Wnt/β-catenin signaling

## Abstract

Epithelial–mesenchymal transition (EMT) and mesenchymal–epithelial transition (MET) are evolutionarily conserved cellular processes defined by reversible conversions between epithelial and mesenchymal phenotypes. As dynamic regulatory programs, they contribute to cell fate determination, tissue remodeling, and functional maturation during embryogenesis. In the cochlea, emerging evidence suggests that EMT/MET are implicated in certain aspects of sensory epithelium development. This review systematically dissects the molecular mechanisms underlying EMT and MET during cochlear development, along with the regulatory networks that control cell fate and signaling pathways. We further explore the emerging functions of these processes in cochlear pathologies, integrating recent advances to clarify their physiological and pathological relevance. By providing a comprehensive synthesis, this work aims to establish a theoretical framework for developing therapeutic strategies against related disorders.

## 1. Introduction

The cochlea, a vital structure within the auditory system, plays a crucial role in sound perception and processing. Its development is marked by intricate cellular differentiation and tissue remodeling processes that ensure the formation of a functional organ capable of translating sound waves into neural signals [1]. The cochlea’s architecture consists of a mosaic of specialized cell types, including sensory hair cells and supporting cells, each with distinct morphologies and functional properties that vary along the cochlea’s tonotopic axis [2]. This diversity is essential for the cochlea’s ability to respond to a wide range of sound frequencies, highlighting the importance of precise cellular development and organization during cochlear maturation [3]. A key aspect of cochlear development is the phenomenon of epithelial–mesenchymal transition (EMT) and its reverse process, mesenchymal–epithelial transition (MET). EMT is characterized by the loss of epithelial cell characteristics and the acquisition of mesenchymal traits, which facilitate processes such as tissue repair, inflammation, and tumor metastasis. Conversely, MET involves the reversion of mesenchymal cells to an epithelial phenotype, which is equally significant during developmental and pathological contexts [4]. These transitions are not merely cellular transformations but are integral to the dynamic remodeling that occurs within the cochlea, influencing both structural integrity and functional capabilities. Ptch1-mediated Shh signaling regulates the basal-to-apical differentiation of marginal cells, which is essential for stria vascularis formation and endocochlear potential maintenance [5].

Similarly, Shh signaling via Gli2 has been shown to regulate the timing of hair cell differentiation by maintaining Sox2 expression in prosensory cells, ensuring proper basal-to-apical patterning during cochlear development [6].

Recent studies have underscored the critical role of the Wnt/β-catenin signaling pathway in regulating the fate of both epithelial and mesenchymal cells within the cochlea [7]. This pathway is essential for orchestrating cellular behaviors that dictate cochlear development and homeostasis. Dysregulation of Wnt signaling can lead to aberrant cellular transitions, contributing to various cochlear pathologies.

The interplay between EMT and MET within the cochlea is not only fundamental to normal development but also has significant implications for understanding cochlear-related diseases. The mechanisms underlying these transitions can provide insights into the pathophysiology of hearing loss and other auditory disorders. By systematically reviewing the molecular pathways and regulatory mechanisms involved in EMT and MET, we aim to enhance our understanding of cochlear biology and pave the way for novel therapeutic strategies targeting cochlear diseases. Given that direct molecular evidence from human temporal bone samples remains limited, this review primarily synthesizes mechanistic insights derived from mouse developmental biology, cochlear organotypic culture models, and in vitro systems. This review seeks to elucidate the intricate balance between these transitions and their physiological and pathological significance, ultimately contributing to advancements in the treatment of cochlear conditions.

## 2. Definition and Biological Characteristics of EMT and MET

EMT is a complex biological process characterized by the transformation of epithelial cells into mesenchymal-like cells. This transition involves a significant loss of epithelial characteristics, including cell polarity and intercellular adhesion, alongside the acquisition of mesenchymal traits that enhance cellular motility and invasiveness [8,9]. EMT is typically triggered by various extracellular signals, including growth factors, cytokines and extracellular matrix components, which activate specific signaling pathways such as TGF-β, Wnt, and Notch. During this process, epithelial cells downregulate key markers such as E-cadherin, which is crucial for maintaining cell–cell adhesion, and upregulate mesenchymal markers like N-cadherin and vimentin, which facilitate cell migration and invasion [10]. Importantly, EMT is not merely a binary switch but rather a spectrum of cellular states, where cells may exhibit a partial EMT phenotype, characterized by the co-expression of both epithelial and mesenchymal markers.

Conversely, MET represents the reverse process, where mesenchymal cells revert to an epithelial phenotype. This transition is crucial for processes such as tissue repair and regeneration, where cells need to regain their epithelial characteristics to restore tissue architecture and function [11]. MET is characterized by the re-establishment of cell polarity, the reformation of tight junctions and the re-expression of epithelial markers like E-cadherin. Similarly to EMT, MET is regulated by a variety of signaling pathways and transcription factors, which can be influenced by the microenvironment and the presence of specific growth factors [12,13]. The dynamic interplay between EMT and MET is essential for maintaining tissue homeostasis and facilitating wound healing, highlighting the importance of these processes in both physiological and pathological contexts [14].

## 3. Spatiotemporal Dynamics of EMT and MET in Cochlear Development

The development of the cochlea is a complex process that involves the interaction between epithelial and mesenchymal cells which are derived from different embryonic origins. During the embryonic stage, the cochlear epithelium and surrounding mesenchymal cells collaborate to form the intricate structures of the cochlea, including the organ of Corti, which houses the sensory hair cells responsible for hearing [15]. The epithelial cells, specifically the sensory precursor cells, undergo terminal divisions and differentiate into both sensory and non-sensory cells, contributing to the functional architecture in the cochlea. Meanwhile, the surrounding mesenchymal cells differentiate to form the cochlear wall, the cochlear axis, and the surrounding cavities, which are essential for maintaining the cochlear structure and function [16]. The dynamic interplay between EMT and MET plays a critical role in regulating cell shape and function during cochlear development [7].

E11.5–E18.5: Neural crest cells originate from the dorsal edge of the neural tube, undergo EMT, and emigrate to migrate to the interstitial region of the cochlea [17]. This process endows them with migratory capacity; neural crest cells primarily differentiate into cochlear mesenchyme (e.g., connective tissue, pericytes) and interstitial components such as intermediate cells of the stria vascularis, thereby providing structural support for cochlear development and participating in microenvironment regulation. Meanwhile, otic neuroepithelial cells derived from the otic placode differentiate into spiral ganglion neuron precursors, which migrate and aggregate to form the spiral ganglion, laying the foundation for cochlear innervation. Cochlear interstitial cells (including those of neural crest origin and locally proliferating cells) further differentiate to refine the structural support function of the cochlea.

The perinatal period (P0–P10) is another critical stage in cochlear development. During this period, mesenchymal cells of the spiral ligament have been proposed to undergo MET-like transitions (inferred from marker shifts and morphological changes, though definitive lineage-tracing evidence for a classic MET origin remains limited) to form the basal cell layer of the stria vascularis [18]. This process involves changes in cell morphology and remodeling of the extracellular matrix (ECM). Matrix metalloproteinases (MMPs) play a crucial role in this process by degrading the ECM and promoting mesenchymal cell migration. The formation of the basal cell layer of the stria vascularis is essential for maintaining the high potassium concentration of the endolymph, which is crucial for normal cochlear function [19].

During the postnatal period (P8–P25), supporting cells of the organ of Corti, including pillar cells and Deiters’ cells, undergo significant morphological and junctional remodeling as part of normal cochlear development. These coordinated changes contribute to the formation of the Corti tunnel and Nuel spaces [20], involving alterations in cell shape and the reorganization of intercellular junctions and ECM For instance, the deposition of laminin and type IV collagen within the basement membrane of the stria vascularis provides structural support during this morphogenetic phase. These cellular transformations exhibit features reminiscent of EMT, such as cytoskeletal reorganization and altered cell adhesion, suggesting a potential mechanistic link to EMT; they represent a programmed developmental process essential for establishing the mature organ of Corti architecture. This remodeling is critical for the mechanotransduction function, determining the precise mechanical coupling between the tectorial membrane and the hair cells.

The dynamic changes in EMT and MET during cochlear development are regulated by various molecular mechanisms. Recent organoid models derived from human pluripotent stem cells have shown that cochlear hair cells can be generated in vitro through timed modulation of SHH and WNT signaling. These cochlear organoids exhibit both inner and outer hair cell-like phenotypes, providing a powerful model for studying human auditory development and EMT-related pathologies [21].

Key transcription factors such as Tbx18 and the Wnt/β-catenin signaling pathway play crucial roles in this process [7,18]. The absence of Tbx18 leads to defects in the basal cell layer of the stria vascularis, affecting the maintenance of the endolymphatic potential. In contrast, overactivation of the Wnt/β-catenin signaling pathway can lead to excessive EMT of the organ of Corti epithelial cells, resulting in the formation of ectopic cysts. Additionally, the remodeling of ECM and the regulation of cell–cell connections are also key factors in the EMT and MET processes. The coordinated action of these molecular mechanisms ensures the cell transformation and functional maturation during cochlear development. Single-cell transcriptomic analysis reveals that outer hair cells continue maturing postnatally until P28, whereas inner hair cells reach maturity earlier, consistent with the temporal regulation of EMT/MET-related structural remodeling [22]. In summary, the spatiotemporal dynamics of EMT and MET during cochlear development are a complex and precise process involving the coordinated action of multiple cell types and molecular mechanisms. These processes contribute to cochlear morphogenesis and play a role in providing the foundation for cochlear functional maturation.

## 4. The Role of Wnt/β-Catenin Signaling Pathway in Cochlear Cell Fate Conversion

The Wnt/β-catenin signaling pathway exerts cell type-dependent and bidirectional effects on cellular plasticity during cochlear development, with features reminiscent of both epithelial–mesenchymal transition (EMT) and mesenchymal–epithelial transition (MET). In the developing cochlea, epithelial cells originate from the otic placode and differentiate into sensory and supporting cells within the cochlear duct, while mesenchymal cells derive from the periotic mesenchyme and give rise to structures such as the spiral ligament, modiolus, and pericochlear spaces. Upon Wnt activation, distinct cellular responses occur in these two lineages.

In epithelial cells, Wnt signaling drives a plastic state with characteristics reminiscent of EMT. This is evidenced by the formation of proliferative cell clusters (foci) that exhibit a shift in marker expression, losing epithelial markers (e.g., E-cadherin, EpCAM, Krt8, Sox2, Prox1, Jag1) and gaining mesenchymal-associated markers (e.g., vimentin, LEF1). This phenotypic shift is accompanied by increased proliferation (evidenced by elevated Ki67, EdU, and Cyclin D1 expression), aligning with features associated with an EMT-like process where epithelial cells adopt a more mesenchymal-like, proliferative state [7]. In other epithelial systems, such as human lens epithelial cells, hyaluronic acid (HA) has been shown to promote EMT via CD44-mediated upregulation of TGF-β2, suggesting a conserved mechanism of EMT regulation involving extracellular matrix components and Wnt-independent pathways [23].

Conversely, in mesenchymal cells, Wnt activation induces a plastic state with features suggestive of MET. This leads to the formation of ectopic pericochlear spaces and cell clusters that exhibit a shift in marker expression, including loss of mesenchymal markers (e.g., Sox9, Pou3f4) and acquisition of epithelial markers (e.g., E-cadherin, ZO1), alongside suppressed proliferation (reflected by reduced Ki67, EdU, and Cyclin D1 expression). This response mirrors features associated with a MET-like process, where mesenchymal cells adopt a more epithelial-like, less proliferative state.

Experimental evidence supporting these findings includes lineage tracing using Axin2-CreERT2 and Lgr5-CreERT2 mice, which revealed that Wnt activation drives distinct cellular behaviors in epithelial (Lgr5+) and mesenchymal (Axin2+) cells. Clonal analysis with multicolor Cre reporters (Confetti/Rainbow) further demonstrated that epithelial foci are predominantly monoclonal, arising from clonal expansion of single cells, whereas mesenchymal foci are multiclonal, formed through cell aggregation rather than proliferation. Immunohistochemistry and RNAscope confirmed the molecular changes associated with these Wnt-induced plastic states.

Collectively, these results highlight the Wnt/β-catenin pathway’s ability to bidirectionally modulate cellular plasticity and fate decisions in a context-dependent manner during cochlear development: promoting an EMT-like epithelial plasticity program in epithelial cells and inducing a MET-like mesenchymal plasticity program in mesenchymal cells. This dual regulatory role underscores the pathway’s precise control over cell state transitions, providing critical insights into cochlear morphogenesis and potential avenues for regenerative therapies targeting hearing loss.

## 5. Cell Lineage Tracing Reveals Differential Cell Fate Under Wnt Signaling

The application of multicolor fate tracing technology has significantly advanced our understanding of the dynamics of cell fate decisions in various tissues, particularly in the context of the Wnt signaling pathway. Recent studies have demonstrated that epithelial cells can undergo clonal expansion, resulting in the formation of distinct cell populations, while mesenchymal cells tend to aggregate into clusters. This phenomenon highlights the role of Wnt signaling in dictating divergent fate pathways among different cell types. For instance, in the context of cranial mesenchyme, Wnt signaling has been shown to mediate the balance between osteogenic and chondrogenic fates, illustrating its pivotal role in lineage specification. Specifically, the activation of Wnt/β-catenin signaling in mesenchymal progenitors leads to the suppression of chondrocyte fate markers while promoting osteoblast differentiation, thereby influencing the overall cellular composition within developing tissues [24]. Furthermore, lineage tracing studies utilizing advanced genetic tools have revealed that Wnt-responsive progenitor cells are crucial for maintaining tissue homeostasis and regenerative capacity. These studies indicate that the presence of Wnt ligands in the microenvironment can significantly alter the fate of progenitor cells, steering them toward specific lineages based on the signaling context. For example, in the context of skeletal development, the loss of Wnt signaling results in ectopic cartilage formation, underscoring the necessity of Wnt signaling for proper lineage commitment [25]. Moreover, the interplay between Wnt signaling and other pathways, such as Notch and Hedgehog, further complicates the regulatory network governing cell fate decisions, as these signals can synergistically or antagonistically influence the outcomes of progenitor cell differentiation. This complexity is evident in studies involving Gli1+ progenitors, where Wnt signaling has been shown to regulate their proliferation and differentiation during bone development and osteoarthritis, highlighting the nuanced role of Wnt in mediating cellular responses to developmental cues [26]. Overall, the insights gained from these lineage tracing studies not only elucidate the mechanisms underlying cell fate determination but also emphasize the importance of Wnt signaling in orchestrating the intricate balance between different cell types during development and regeneration.

## 6. The Potential Pathological Significance of EMT and MET in Cochlear Diseases

### 6.1. Abnormalities in EMT/MET and Cochlear Development Defects

EMT and MET are important biological processes in cochlear development and homeostasis. Among these, the signaling pathway mediated by hepatocyte growth factor (HGF) and its receptor MET plays a significant role in cochlear development. This pathway exerts a notable influence on the migration and differentiation of cells within the cochlea. When this pathway is disrupted, it may lead to developmental defects in the stria vascularis—a key structure that maintains the ionic composition of the endolymph and the electrochemical gradients required for hair cell function—whose abnormal development directly impairs auditory function [27].

In mouse models, HGF deficiency leads to the failure of neural crest cells to migrate into the stria vascularis, resulting in reduced endocochlear potential and ultimately severe hearing loss. These findings indicate that the dynamic balance between EMT and MET is of great importance for the structural integrity and functional maintenance of the cochlea; deviations from this balance may lead to abnormal structural and functional alterations, manifested as hearing loss.

### 6.2. The Role of EMT in Cochlear Fibrosis

Excessive accumulation of extracellular matrix (ECM) components, such as collagen and glycosaminoglycans, contributes to structural and functional alterations in cochlear tissue. In the context of cochlear fibrosis—typically observed after implantation or trauma—histopathological studies consistently demonstrate that fibroblast proliferation and activation drive pathological changes primarily within the perilymphatic spaces: the scala tympani, scala vestibuli, and other associated compartments. These processes lead to fibrous tissue deposition, neovascularization, and often new bone formation, which collectively result in partial or complete obliteration of the scalae. Such remodeling disrupts perilymph circulation, compromises nutrient/waste exchange for the organ of Corti, and may exert mechanical pressure on the delicate structures of the cochlear duct, ultimately impairing hair cell survival and function and exacerbating hearing loss [28,29]. Additionally, inflammatory cells, including macrophages and lymphocytes, are closely associated with pathological changes in the cochlea. Their activation not only induces local inflammatory responses but may also further promote fibrotic progression [30]. EMT has been implicated as a mechanism contributing to cochlear fibrosis, driving pathological remodeling that compromises hearing and cochlear implant function [31,32]. In addition to fibrosis, EMT-related signaling disruptions during cochlear maturation can lead to stereocilia defects in inner hair cells. For instance, deletion of Jagged1 (JAG1), a Notch ligand, during cochlear maturation results in fused and elongated stereocilia, leading to auditory neuropathy-like hearing loss without affecting outer hair cells [33].

Following trauma such as electrode insertion or immune challenge, mesothelial cells lining the scala media can undergo EMT, losing epithelial markers like E-cadherin and acquiring a myofibroblastic phenotype marked by α-smooth muscle actin expression and deposition of extracellular matrix (ECM) components including collagen I, fibronectin, and laminin. This phenotypic conversion was directly demonstrated in vivo via adenoviral GFP tracing, revealing migration of transformed mesothelial cells into fibrotic regions where they contributed to scar formation.

Functionally, fibrosis—a poly-cellular process driven by multiple cell types including fibroblasts, macrophages, and potentially EMT-derived myofibroblasts—leads to scala tympani obliteration, compression of spiral ganglion neurons and hair cells, and subsequent hearing threshold elevations exceeding 60 dB as measured by auditory brainstem responses. Notably, EMT represents one possible mechanism contributing to this fibrotic response, though it is not the sole or dominant driver.

The resulting fibrotic tissue reaction, which is primarily responsible for increased impedance around cochlear implant electrodes, encapsulates the electrodes and elevates impedance. This impedance rise reduces stimulation efficacy and battery longevity. While EMT may participate in the broader fibrotic cascade, attributing impedance changes specifically to EMT-driven fibrosis remains speculative; the alterations are chiefly associated with the generalized fibrotic and inflammatory tissue reaction [34].

Therapeutic interventions targeting this EMT pathway have shown distinct efficacy profiles. Dexamethasone, while effective in suppressing the inflammatory cytokine IL-1β, incompletely inhibits TGF-β1 signaling and demonstrates concentration-dependent toxicity, with high concentrations causing significant sensory cell loss. In contrast, the antimitotic agent cytarabine (Ara-C) directly inhibits myofibroblast proliferation with greater potency and markedly reduces TGF-β1 secretion without compromising sensory cell survival. In animal models, Ara-C has been shown to significantly reduce fibrosis and better preserve auditory function compared to dexamethasone, establishing a superior risk-benefit ratio [28].

Collectively, these findings suggest EMT as one of several mechanisms contributing to cochlear fibrosis and highlight cytarabine as a promising therapeutic strategy targeting this pathway. However, it should be noted that the degree and prevalence of bona fide EMT in local epithelial populations (e.g., mesothelial cells) within the human or mouse cochlea remain incompletely defined, and fibrosis likely arises from synergistic interactions between EMT, inflammation, fibroblast activation, and other processes. Future development of localized drug delivery systems—such as drug-eluting electrode arrays—could enable targeted suppression of EMT while minimizing systemic exposure, offering a clinically viable approach to preserve residual hearing and optimize cochlear implant performance.

### 6.3. The Potential of EMT/MET as Therapeutic Targets

EMT and its reverse process, MET, are critical biological processes that have garnered significant attention as potential therapeutic targets in cochlear diseases, including developmental abnormalities. The regulation of signaling pathways, particularly Wnt, plays a pivotal role in modulating the EMT/MET processes. Wnt signaling, for instance, has been implicated in the maintenance of epithelial characteristics and the regulation of cell fate decisions during cochlear development. By targeting Wnt pathway components, it may be possible to enhance MET, thereby promoting the restoration of epithelial integrity and function in the cochlea.

By leveraging insights gained from studies on EMT/MET regulation, clinicians and researchers can explore novel treatment paradigms that not only address the symptoms of cochlear disorders but also target the underlying cellular transitions contributing to disease pathology. This dual approach may lead to more effective interventions that restore normal function and improve quality of life for patients suffering from conditions related to aberrant EMT/MET processes in the cochlea.

## 7. Multidisciplinary Approaches to Promote Cochlear Cell Fate Research

The exploration of cochlear cell fate has significantly advanced through the integration of various scientific disciplines, including developmental biology, molecular biology, and clinical medicine. Developmental biology provides a foundational understanding of the processes that govern cell differentiation and organization within the cochlea. For instance, research has elucidated the roles of transcription factors such as Sox9 and Atoh1 in determining the fate of cochlear cells, highlighting how the regulation of these factors can influence the balance between hair cells and supporting cells [35]. Molecular biology complements this understanding by employing techniques such as single-cell RNA sequencing and epigenomic profiling to dissect the intricate regulatory networks that dictate cell fate decisions. This approach allows researchers to identify critical signaling pathways and gene expression patterns that are essential for cochlear development and regeneration [36]. Furthermore, clinical medicine plays a vital role by translating these findings into therapeutic strategies aimed at treating hearing loss, a condition often resulting from the loss of cochlear hair cells. For example, the application of gene therapy to induce the expression of hair cell-specific transcription factors has shown promise in promoting hair cell regeneration in animal models [37,38]. Additionally, the use of bioinformatics and systems biology has emerged as a powerful tool to integrate and analyze large datasets from various studies, facilitating a more comprehensive understanding of cochlear cell fate regulation. Recent advances in data-driven modeling and single-cell trajectory analysis have revealed that EMT proceeds through multiple intermediate states, challenging the traditional binary E-M model. These approaches, including pseudotime inference and RNA velocity, provide new insights into the dynamic paths of EMT in both developmental and pathological contexts [39]. By employing computational models, researchers can simulate the dynamic interactions between different cell types and their microenvironment, thereby predicting how changes in signaling pathways may affect cochlear development and function [40]. This multidisciplinary approach not only enhances our understanding of the basic biological processes involved but also identifies potential targets for intervention in hearing loss. For instance, the identification of key regulatory genes and their interactions can inform the development of pharmacological agents or gene therapies that aim to restore or enhance cochlear function [41,42]. Moreover, collaboration among researchers from diverse fields fosters innovation and accelerates the translation of basic research into clinical applications. The establishment of interdisciplinary research teams that include developmental biologists, molecular biologists, clinicians, and bioinformaticians can lead to the development of novel therapeutic strategies that leverage the strengths of each discipline. For example, combining insights from developmental biology with advanced molecular techniques can lead to the identification of new biomarkers for cochlear cell types, which can be used to monitor the efficacy of regenerative therapies in clinical settings [43].

Integrating RNA velocity with cochlear single-cell datasets—such as the mouse aging atlas or human inner-ear organoid data—enables real-time tracking of EMT/MET velocity vectors [44,45]. For example, during aging the Tgfb1/Mmp13 velocity vector in stria vascularis intermediate cells points toward fibrosis before histological fibrosis appears, whereas in early development the Atoh1 velocity in Sox2+ progenitor cells points toward hair-cell fate, mirroring the Twist1-driven EMT vector reported in cancer metastasis. Note that the dense otic capsule can introduce dissociation stress; combining cold-enzyme digestion with scVelo’s dynamical model effectively minimizes this technical noise [34,46].

In conclusion, the integration of developmental biology, molecular biology, clinical medicine, and computational approaches is essential for advancing our understanding of cochlear cell fate and developing effective strategies for treating hearing loss. By fostering collaboration across disciplines, researchers can uncover new insights into the mechanisms underlying cochlear development and regeneration, ultimately leading to improved therapeutic options for patients suffering from auditory disorders.

## 8. Conclusions

This review focuses on mechanistic insights derived from murine developmental biology, cochlear organoid models, and in vitro systems. While EMT/MET-associated pathologies (e.g., fibrosis, dysplasia) are clinically relevant, direct molecular evidence from human temporal bone specimens remains limited. Emerging evidence suggests that EMT and MET may serve as promising conceptual frameworks for understanding aspects of cochlear morphogenesis. While these processes are not yet firmly established as central to inner ear development, they offer a valuable lens through which to investigate the dynamic cellular rearrangements required for building the cochlea. Similarly, the Wnt/β-catenin signaling pathway has been implicated in regulating cell state changes in various developmental contexts, including potentially both EMT and MET-like events in the cochlea. Its apparent ability to influence these transitions underscores the complex equilibrium cells must navigate.

However, it is critical to acknowledge that the role of EMT/MET in cochlear development remains an emerging hypothesis. Many aspects—including their precise extent, timing, cell-type specificity, and conservation across species—are supported by limited in vivo evidence, mixed model systems, and partial marker analyses. Future research must prioritize clarifying these fundamental questions. A multifaceted approach, integrating advanced imaging, single-cell genomic profiling, and computational modeling, will be essential to experimentally validate these concepts. By systematically dissecting these processes, we can determine whether EMT/MET are indeed pivotal regulators of cochlear development and function, opening new avenues for understanding and treating auditory disorders.

## Data Availability

No new data were created or analyzed in this study.

## Abbreviations

The following abbreviations are used in this manuscript:

EMT	Epithelial–Mesenchymal Transition
MET	Mesenchymal–Epithelial Transition
Wnt/β-catenin	Wnt/β-catenin signaling pathway
Shh	Sonic Hedgehog
Gli2	GLI family zinc finger 2
Sox2	SRY-box transcription factor 2
Tbx18	T-box transcription factor 18
Pou3f4	POUs domain, class 3, transcription factor 4
JAG1	Jagged 1
HGF	Hepatocyte Growth Factor
MET (receptor)	MET proto-oncogene, receptor tyrosine kinase
ECM	Extracellular Matrix
MMPs	Matrix Metalloproteinases
Ki67	Ki67 antigen
EdU	5-ethynyl-2′-deoxyuridine
Cyclin D1	Cyclin D1
CD44	Cluster of Differentiation 44
TGF-β	Transforming Growth Factor-beta
LEF1	Lymphoid Enhancer-Binding Factor 1
PD0325901	PD0325901 (MEK/ERK inhibitor)
Notch	Notch signaling pathway
HA	Hyaluronic Acid
Axin2	Axis inhibition protein 2
Lgr5	Leucine-rich repeat-containing G-protein-coupled receptor 5
Confetti/Rainbow	Confetti/Rainbow (lineage tracing system)
NDP-KO	Norrie Disease Protein Knockout
Dexamethasone	Dexamethasone
Cytarabine (Ara-C)	Cytarabine (Ara-C)
IL-1β	Interleukin-1 beta
TGF-β1	Transforming Growth Factor-beta 1
KGF	Keratinocyte Growth Factor
p63	TP63 gene product
Sox9	SRY-box transcription factor 9
Atoh1	Atonal bHLH transcription factor 1
Hey1/HeyL	Hairy/enhancer-of-split related with YRPW motif 1/2
Foxg1	Forkhead box G1
Gata3	GATA binding protein 3
TBX2	T-box transcription factor 2
Lrrn1	Leucine-rich repeat neuronal protein 1
